# Effects of long-term metformin intake on postoperative clinicopathological characteristics in patients with invasive lung adenocarcinoma and type 2 diabetes mellitus: A retrospective analysis

**DOI:** 10.1371/journal.pone.0329277

**Published:** 2025-07-30

**Authors:** Xiaowen Zhang, Jingwei Liu, Ziwei Zhao, Jie Jiang, Guojun Geng

**Affiliations:** 1 Department of Thoracic Surgery, The First Affiliated Hospital of Xiamen University, School of Medicine, Xiamen University, Xiamen, China; 2 Department of Thoracic Surgery, Haicang Hospital of Xiamen, Xiamen, Fujian Province, China; 3 Pathology Department, The First Affiliated Hospital of Xiamen University, School of Medicine, Xiamen University, Xiamen, China; 4 Department of Thoracic Surgery, The First Affiliated Hospital of Xiamen University, Xiamen, China; University of the Punjab Quaid-i-Azam Campus: University of the Punjab, PAKISTAN

## Abstract

**Objective:**

This retrospective study aimed to evaluate whether long-term metformin use is associated with less aggressive clinicopathological characteristics in patients with invasive lung adenocarcinoma (LUAD) and type 2 diabetes mellitus (T2DM).

**Methods:**

We reviewed patients with both invasive LUAD and T2DM who underwent curative lung cancer resection and lymph node dissection between January 2012 and August 2022. Patients were divided into a metformin group and a non-metformin group based on their antidiabetic treatment. Clinicopathological outcomes included tumor size, TNM stage, histologic differentiation, Ki-67 expression, and pathological subtypes. Comparisons were made using Student’s t-test, Mann–Whitney U test, χ² test, or Fisher’s exact test, as appropriate.

**Results:**

A total of 130 patients were included (45 metformin users and 85 non-users), with no significant differences in baseline characteristics. The metformin group showed smaller tumors (1.78 ± 0.87 cm vs. 2.21 ± 1.28 cm; *p* = 0.049), fewer cases of high Ki-67 expression (>15%) (35.6% vs. 62.3%; *p* = 0.004), and no lymph node metastasis (0% vs. 15.3%; *p* = 0.022). Additionally, patients on metformin had better differentiation (*p* = 0.039) and earlier TNM stages (*p* = 0.03).

**Conclusion:**

Long-term metformin use in diabetic patients with invasive LUAD was associated with more favorable clinicopathological features, including smaller tumor size, lower proliferative index, and absence of nodal metastasis. These findings support a potential anti-tumor role of metformin in lung adenocarcinoma.

## 1 Introduction

Lung cancer remains the leading cause of cancer-related mortality worldwide, with adenocarcinoma being its most common histological subtype [1,2]. According to the WHO classification and multiple institutional studies, approximately 80–90% of resected lung adenocarcinomas are classified as invasive LUAD [3–5]. The increasing use of low-dose chest CT has led to earlier detection of lung cancers, including among younger patients. Meanwhile, the global prevalence of type 2 diabetes mellitus (T2DM) is rising, particularly in middle-aged and elderly populations [6–9]. As a result, comorbid LUAD and T2DM are frequently encountered in clinical practice.

Metformin, a first-line oral antidiabetic drug, has drawn attention for its potential anti-cancer effects. Previous studies have shown that metformin use is associated with reduced risk or improved prognosis in various malignancies, including colorectal, breast, and genitourinary cancers [10–12]. Emerging evidence also suggests a protective association between metformin and lung cancer risk. For instance, Zhang et al. reported that metformin use was linked to a significantly lower incidence of lung cancer in diabetic patients [1]. Tsai MJ et al. found that metformin reduces the risk of lung cancer in patients with type 2 diabetes mellitus in a dose-dependent manner [2].

While several studies have evaluated metformin’s role in lung cancer risk and survival, less is known about its impact on tumor biology and pathological features, particularly in the context of surgically resected invasive LUAD. Invasive LUAD was selected for this study because of its predominance among early-stage resectable tumors and the availability of comprehensive pathological assessment post-resection, enabling reliable evaluation of tumor aggressiveness indicators.

This study aimed to assess whether long-term metformin use is associated with less aggressive clinicopathological features—such as tumor size, Ki-67 expression, degree of differentiation, TNM stage, and nodal involvement—in patients with T2DM and invasive LUAD who underwent curative surgery.

## 2 Materials and methods

### 2.1 Study design and data source

We retrospectively analyzed the clinical data of patients with invasive lung adenocarcinoma (LUAD) and type 2 diabetes mellitus (T2DM) who underwent radical surgical resection and lymph node dissection between January 2012 and August 2022 at the First Affiliated Hospital of Xiamen University, Xiamen, China. Clinical information was retrieved from the hospital’s electronic medical record system. Two independent researchers extracted data, and discrepancies were resolved by discussion with a third reviewer. The study protocol was approved by the Medical Ethics Committee of the First Affiliated Hospital of Xiamen University. The requirement for informed consent was waived due to the retrospective nature of the study. Data collection was conducted between 5 May 2024 and 15 June 2024. This study complied with the principles of the Declaration of Helsinki.

### 2.2 Participants and grouping

**Fig 1:**
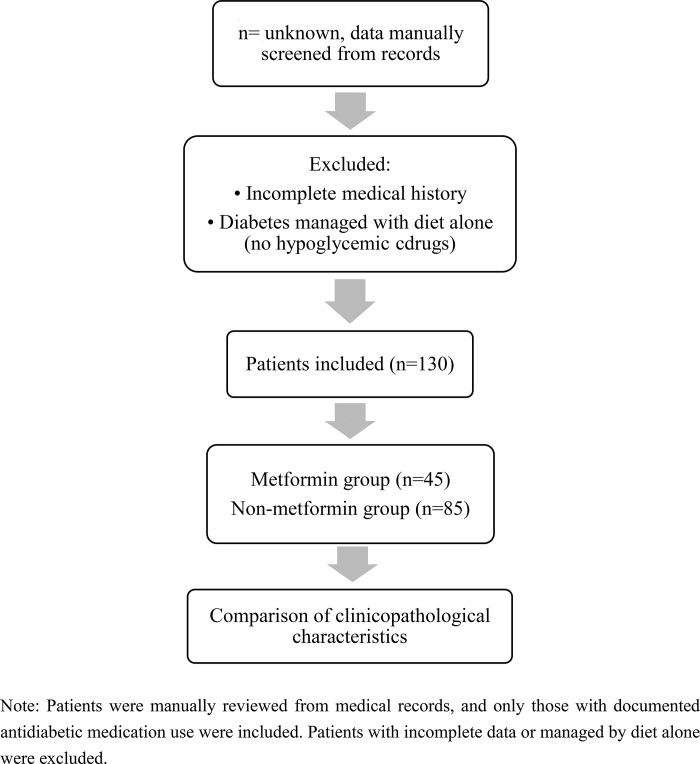
Enrollment process for this study.

A total of 130 patients were included, comprising 67 men and 63 women, with a mean age of 64 years (range: 39–84). All patients underwent anatomical lobectomy and systematic lymph node dissection. Based on their antidiabetic regimens, patients were categorized into two groups: the metformin group (n = 45), who had taken metformin continuously for more than 3 months prior to surgery, and the non-metformin group (n = 85), who had used other hypoglycemic agents or insulin for at least 3 months without metformin (Fig 1). The definition of long-term metformin use as >3 months was based on previous studies, including a South Korean study on cancer risk and a clinical trial assessing immunotherapy outcomes in non-small cell lung cancer (NSCLC) [3].

### 2.3 Variables and definitions

The following clinicopathological variables were collected: age, sex, family history of cancer, comorbidities, tumor size, TNM stage (8th edition), tumor differentiation grade, Ki-67 proliferation index, and pathological subtypes. Tumor size was measured on preoperative CT scans as the average of the maximum transverse diameter and its perpendicular short axis in the lung window. The Ki-67 index was assessed using immunohistochemical staining of formalin-fixed, paraffin-embedded surgical specimens. All samples were fixed in 10% neutral buffered formalin via pleural and bronchial injection, followed by hematoxylin-eosin (H&E) and Ki-67 immunohistochemical staining. Ki-67 values were extracted from official pathology reports, with a cutoff value of >15% defined as high Ki-67 expression. Tumor differentiation grade and pathological subtypes were documented based on histopathological findings as recorded in the electronic medical records.

### 2.4 Statistical analysis

Continuous variables were first tested for normality. Normally or approximately normally distributed variables were presented as mean ± standard deviation (SD), and compared using Student’s t-test. Non-normally distributed variables were expressed as median [interquartile range, IQR], and compared using the Mann–Whitney U test. Categorical variables were presented as counts (percentages), and compared using the chi-square test or Fisher’s exact test, as appropriate. All statistical analyses were conducted using SPSS 25.0. A p-value < 0.05 was considered statistically significant.

## 3 Results

### 3.1 Demographic characteristics of patients in the metformin and non-metformin groups

Baseline characteristics between the metformin (n = 45) and non-metformin (n = 85) groups were comparable in terms of sex, age, BMI, diabetes duration, smoking, alcohol consumption, and comorbidities including hypertension, coronary heart disease, stroke, and COPD (all *p* > 0.05; Table 1).

**Table 1:** Demographic characteristics of patients in the metformin and non-metformin groups.

Variable	Metformin group (n = 45)	Non-metformin group (n = 85)	Statistical Value	*p*
Sex			3.145	0.076
Male	28 (62.2%)	39 (45.9%)		
Female	17 (37.8%)	46 (51.1%)		
Age (y, x ± s)	63.73 ± 8.64	63.98 ± 8.10	t = −0.159	0.874
BMI (kg/cm^2^, x ± s)	23.87 ± 2.46	23.78 ± 3.27	t = −0.176	0.861
Diabetes (y, M [P25, P75])	8.56 (3.50, 10.00)	8.12(1.50, 10.00)	Z = −1.780	0.075
Drinking history			0.624	0.430
Yes	5 (11.1%)	6 (7.1%)		
No	40 (88.9%)	79 (92.9%)		
Smoking history			2.157	0.124
Yes	19 (42.2%)	25 (29.4%)		
No	26 (57.8%)	60 (70.6%)		
Other chronic disease			0.013	0.908
Yes	26 (57.8%)	50 (58.8%)		
No	19 (42.2%)	35 (41.2%)		

### 3.2 Comparison of Ki-67 status between the metformin and non-metformin groups

In the metformin group, 29 cases (64.4%) had a Ki-67 index > 15% while in the non-metformin group, 32 cases (37.7%) had a Ki-67 index ≤ 15%. The difference between the two groups was statistically significant (>15%: 35.6% vs. 62.3%; RR = 0.57, 95% CI: 0.42–0.77, *p* = 0.004; Table 2).

**Table 2:** Comparison of Ki-67 status between the metformin and non-metformin groups.

	Metformin group	Non-metformin group	*χ* ^*2*^	*p*
n	45	85	8.484	0.004
Ki-67 ≤ 15%	29 (64.4%)	32 (35.6%)		
Ki-67 > 15%	16 (37.7%)	53 (62.3%)		

### 3.3 Comparison of lymph node metastasis between the metformin and non-metformin groups

The number of N0, N1, N2, and N3 lymph nodes metastases cases in the metformin group was 45 (100%), 0, 0, and 0, respectively, and in the non-metformin group was 72 (84.7%), 8 (9.4%), 5 (5.9%), and 0, respectively. There was a statistically significant difference between the two groups (0% vs. 15.3%, *p* < 0.05; Table 3).

**Table 3:** Comparison of lesions stages between the metformin and non-metformin groups.

	Metformin group (n = 45)	Non-metformin group (n = 85)	*χ* ^*2*^	*p*
N0	45 (100.00)	72 (84.7%)	7.647	0.022
N1	0	8 (9.4%)		
N2	0	5 (5.9%)		
N3	0	0		

### 3.4 Comparison of the longest diameters of the tumor, tumor site, tumor-differentiation, T stage, and TNM stage between the metformin and non-metformin groups

Tumor size was significantly smaller in the metformin group than in the non-metformin group (1.78 ± 0.87 vs. 2.21 ± 1.28 cm, MD=−0.43, 95% CI: −0.80 to −0.06, *p* < 0.05). No statistically significant difference was found in lesion location between the two groups. The number of cases with well, moderately, and poorly differentiated tumors was 12 (26.7%), 30 (66.7%), and 3 (6.6%), respectively, in the metformin group and 10 (11.8%), 60 (70.6%), and 15 (17.6%), respectively, in the non-metformin group. The difference between the two groups was statistically significant (*p* < 0.05). The numbers of patients with T1, T2, T3, and T4 lesions in the metformin group were 40 (88.9%), 5 (11.1%), 0, and 0, respectively. The numbers of patients with T1, T2, T3, and T4 in the non-metformin group were 61 (71.8%), 20 (23.5%), 3 (3.5%), and 1 (1.2%), respectively. The difference between the two groups was statistically significant (*p* < 0.05). The number of patients in the metformin group in the IA1, IA2, IA3, IB, IIA, IIB, IIIA, and IVA stages of the disease was 7 (15.6%), 26 (57.8%), 7 (15.6%), 3 (6.6%), 2 (4.4%), 0, 0, and 0, respectively. Those in the non-metformin group were 14 (16.5%), 31 (36.5%), 11 (12.9%), 12 (14.1%), 1 (1.2%), 10 (11.8%), 5 (5.8%), and 1 (1.2%), respectively. The difference between the two groups was statistically significant (P < 0.05). Tumor location showed no significant difference (*p* = 0.724), but differentiation grade differed significantly (*p* = 0.039). (Table 4).

**Table 4:** Comparison of tumor characteristics between the metformin and non-metformin groups.

	Metformin group (n = 45)	Non-metformin group (n = 85)	Statistical Value	*p*
Maximal diameter (cm x ± s)	1.784 ± 0.866	2.207 ± 1.278	*t* = −1.988	0.049
Lesion location			*X*^*2*^ = 1.967	0.724
Right upper lobe	18 (40.0%)	27 (31.8%)		
Right middle lobe	4 (8.9%)	7 (8.2%)		
Right lower lobe	5 (11.1%)	17 (20.0%)		
Left upper lobe	11 (24.4%)	21 (24.7%)		
Left lower lobe	7 (15.6%)	13 (15.3%)		
Tumor-differentiation			*X*^*2*^ = 6.488	0.039
Well	12 (26.7%)	10 (11.8%)		
Moderately	30 (66.7%)	60 (70.6%)		
poorly	3 (6.6%)	15 (17.6%)		
T stage			*H* = 5.206	0.023
T1	40 (88.9%)	61 (71.8%)		
T2	5 (11.1%)	20 (23.5%)		
T3	0	3 (3.5%)		
T4	0	1 (1.2%)		
TNM stage			*H* = 4.659	0.031
IA1	7 (15.6%)	14 (16.5%)		
IA2	26 (57.8%)	31 (36.5%)		
IA3	7 (15.6%)	11 (12.9%)		
IB	3 (6.6%)	12 (14.1%)		
IIA	2 (4.4%)	1 (1.2%)		
IIB	0	10 (11.8%)		
IIIA	0	5 (5.8%)		
IVA	0	1 (1.2%)		

### 3.5 Comparison of pathological subtypes between the metformin and non-metformin groups

The number of patients in the metformin group with pathological subtypes including adnexal, alveolar, papillary, micropapillary, solid, complex glandular, sieve, and mucinous subtypes was 31 (68.9%), 32 (71.1%), 18 (40.0%), 7 (15.6%), 13 (28.9%), 0, 0, and 6 (13.3%), respectively. Those in the non-metformin group were 73 (85.9%), 74 (87.1%), 53 (62.4%), 27 (31.8%), 11 (12.9%), 5 (5.9%), 3 (3.5%), and 6 (7.1%), respectively. The differences in the adherent, glandular vesicular, papillary, micropapillary and solid types were statistically significant between the two groups (*p* < 0.05). The differences in the rarer and more malignant complex glandular, septate, and mucinous types were not statistically significant between the two groups (*p* > 0.05) (Table 5).

**Table 5:** Comparison of pathological subtypes between the metformin and non-metformin groups.

	Metformin group (n = 45)	Non-metformin group (n = 85)	*χ* ^*2*^	*p*
Adherent type			5.310	0.021
Yes	31 (68.9%)	73 (85.9%)		
No	14 (31.1%)	12 (14.1%)		
Alveolar type			4.971	0.033
Yes	32 (71.1%)	74 (87.1%)		
No	13 (28.9%)	11 (12.9%)		
Papillary type			5.931	0.015
Yes	18 (40.0%)	53 (62.4%)		
No	27 (60.0%)	32 (37.6%)		
Micropapillary subtype			4.003	0.045
Yes	7 (15.6%)	27 (31.8%)		
No	38 (84.4%)	58 (68.2%)		
Solid type			4.971	0.026
Yes	13 (28.9%)	11 (12.9%)		
No	32 (71.1%)	74 (87.1%)		
Complex glandular body type			2.753*	0.163
Yes	0	5 (5.9%)		
No	45 (100%)	80 (94.1%)		
Sieve type			1.626*	0.551
Yes	0	3 (3.5%)		
No	45 (100%)	82 (96.5%)		
Mucinous type			1.383	0.240
Yes	6 (13.3%)	6 (7.1%)		
No	39 (86.7%)	79 (92.9%)		

## 4 Discussion

Metformin is one of the commonly used oral hypoglycemic agents in clinical practice. The mechanism underlying its effect is the inhibition of glucose production in the liver while increasing the uptake and utilization of glucose by the skeletal muscle [4]. In recent years, various studies have shown that metformin has anti-tumor effects, such as cell cycle blockade, in addition to its hypoglycemic effects [5,6]. Metformin exerts its anti-tumor effects by inducing abnormalities in the energy metabolism of tumor cells [7], inhibiting epithelial-mesenchymal transition [8–10], regulating cellular senescence signals, and selectively killing cancer stem cells, etc. [11–13]. It can also fight tumors cells through various immunomodulatory mechanisms including enhancing the anti-cancer effect of cytotoxic T cells, influencing macrophage polarization, and promoting NK cell activation [14,15]. A study published in Nature used a orthotopic Lewis lung cancer transplant model and found that metformin inhibits lung cancer metastasis by activating AMPK [16]. Lu et al. found that metformin can inhibit the proliferation of NSCLC cells and exert antitumor effects in an AMPK-CEBPB-PDL1 signaling-dependent manner [17]. Zhang et al. showed that metformin antagonizes Ni-refined smoke-induced aerobic glycolysis through AMPK/GOLPH3, thereby exhibiting anticancer properties [18]. Metformin induces AMPK activation mediated by liver kinase B1 (LKB1), thereby inhibiting the mTOR signalling pathway. This effect has been observed in various cancers, including lung cancer. Secondly, metformin negatively regulates mTOR activity after activating AMPK [19]. Mechanistic studies suggest that metformin inhibits lung tumour development by reducing plasma IGF-I levels and decreasing receptor tyrosine kinase signalling [20].

Clinically, there are few patients with lung cancer combined with type 2 diabetes. This could be explained by the increasing detection rate of early-stage lung cancer. The main factors affecting the prognosis of lung cancer are tumor size [21], pulmonary imaging features [22], pathology types [23], pathological subtypes [24], immunohistochemical indexes and staging, etc. [25]. In this study, the maximum diameter of pulmonary nodules was 1.784 ± 0.866 cm in the metformin group and 2.207 ± 1.278 cm in the non-metformin group. The difference was statistically significant. The diameter of a lung nodule can affect the degree of infiltration and stage of lung cancer, which subsequently affects the prognosis of patients with lung cancer [26,27]. This suggests that patients receiving metformin had more favorable pathological features in terms of tumor size. A meta-analysis of 566,435 people with diabetes found that metformin use reduced cancer risk and was negatively associated with lung cancer risk [28]. Lu et al. reported that metformin inhibited the proliferation of non-small cell lung cancer cells via the AMPK-CEBPB-PDL1 pathway in an in vitro experiment [29]. The above studies show that the use of metformin by patients with diabetes can inhibit the proliferation of lung cancer cells to achieve anti-tumor effects.

**Fig 2:**
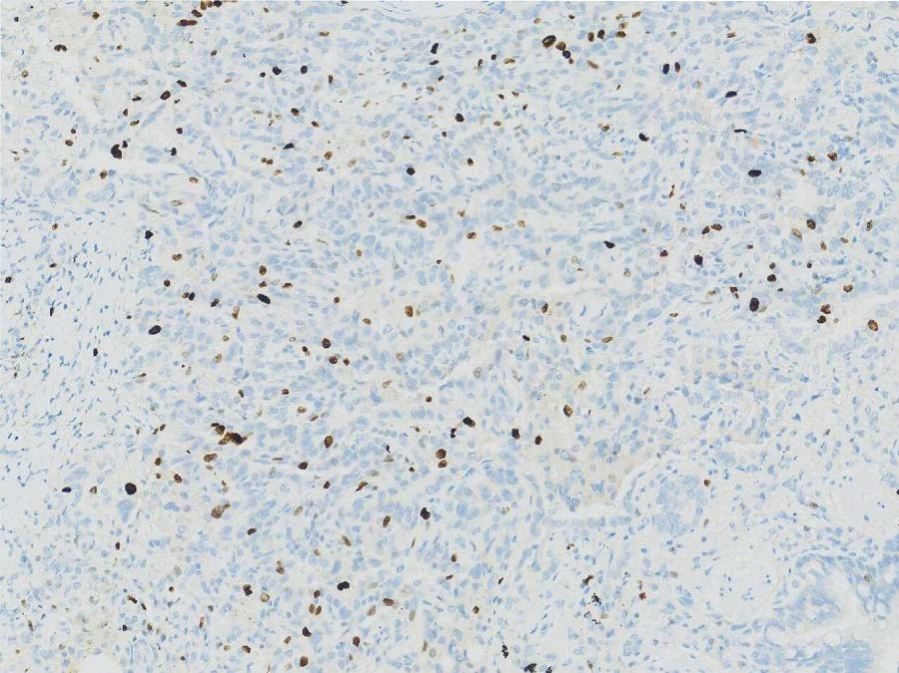
Ki-67 IHC (15% positivity) in metformin group (MIB-1 antibody, × 20).

**Fig 3:**
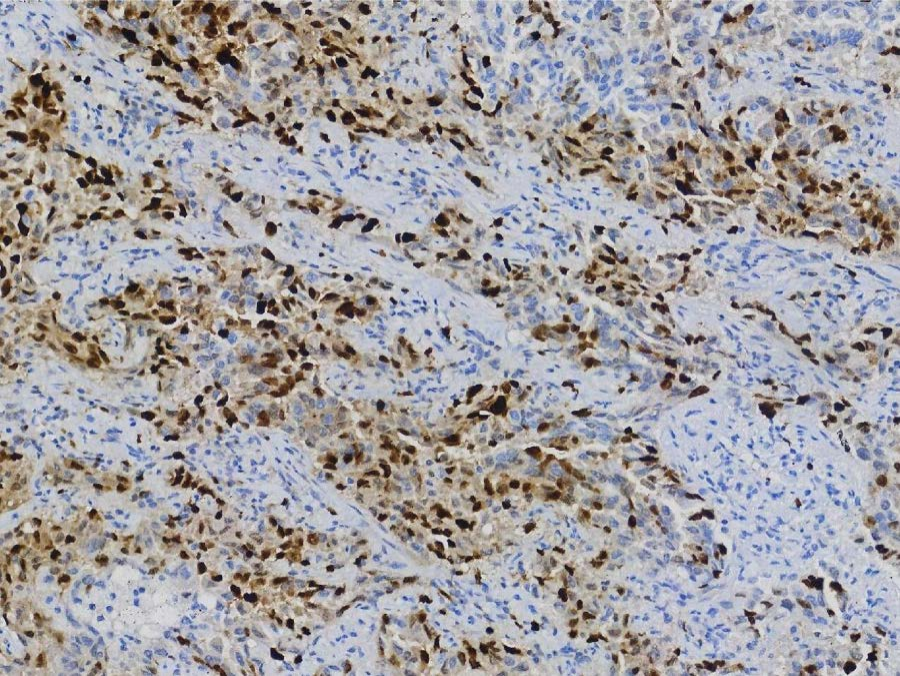
Ki-67 IHC (40% positivity) in non-metformin group (MIB-1 antibody, × 20).

Adenocarcinoma is the most common type of lung cancer pathology. The pathological type of all the 130 patients included in this study was invasive LUAD and most of the patients had stage IA tumors. All the 45 patients in the metformin group had no lymph node metastasis. Eight patients and five patients in the non-metformin group had N1 and N2 lesions, respectively. The difference between the two groups was statistically significant, indicating that regional lymph node metastasis, which worsens the prognosis of patients with LUAD, was less likely to occur in the metformin group compared with the non-metformin group. The metformin group exhibited earlier T and TNM stages compared to the non-metformin group. While these findings may suggest a potential association between metformin use and less aggressive tumor characteristics, the clinical implications of these differences remain to be fully elucidated in studies with long-term follow-up data. Ki-67 is widely recognized as a marker of cell proliferation. It is closely associated with tumor recurrence and metastasis [30]. Patients with high Ki-67 expression tend to have a high probability of metastasis and recurrence [31]. According to previous studies, the Ki-67 status can be divided into two groups, with 15% as the threshold value (the ≤ 15% and > 15% groups). The results in this study showed that there was a statistically significant difference in the Ki-67 status between the two groups. Patients in the non-metformin group compared with the metformin group had higher Ki-67 expression and more active tumor cells (Figs 2 and 3) [32]. Patients in the non-metformin group compared with the metformin group had a higher risk of metastasis and recurrence after surgery.

While several studies have reported anti-tumor effects of metformin, others have shown null or inconsistent results. This heterogeneity may be attributed to differences in study design, tumor types, stages, treatment regimens, or patient characteristics. Our study focused on early-stage invasive LUAD in a surgical cohort, which may partly explain the favorable pathological associations observed. Further research is needed to clarify these divergent findings.

However, since survival outcomes were not available in the current dataset, we cannot directly conclude whether these pathological differences translate into improved long-term prognosis. Further prospective studies with survival analysis are warranted.

Invasive LUAD can also be categorized into different pathological subtypes. The more common of which are adherent, alveolar, papillary, micropapillary, and solid subtypes. Complex glandular, septate, and mucinous subtypes are relatively rare and have higher malignancy. Qu et al. classified tumors into low-risk, moderate-risk, and high-risk groups according to their different subtypes. Low-risk tumors include tumors that are predominantly adnexal and do not have components of the micropapillary and solid subtypes. Moderate-risk tumors are predominantly papillary or alveolar and have no micropapillary or solid subtype components. High-risk tumors are mainly micropapillary or solid subtypes [33]. The pathological subtypes of early-stage lung cancer are mainly the adherent and alveolar subtypes. Patients with micropapillary and solid subtypes have poor prognosis, which correlate strongly with lymph node metastasis [34]. Patients in the high-risk group had a 5-year disease-free survival rate of 71% and high recurrence rate [35]. In the present study, the two groups had statistically significant differences in the following five pathological subtypes: adherent, alveolar, papillary, micropapillary, and solid subtypes. This suggests that patients in the non-metformin group compared with those in the metformin group are more likely to have pathological subtypes that are in the moderate to high-risk group. In this study, there was no statistically significant differences in complex glandular, sieve, and mucinous subtypes. This could be related to the low occurrence of these pathological subtypes and the small sample size. In pathology, LUAD is graded histologically as grade 1 (well-differentiated), grade 2 (moderately-differentiated), and grade 3 (poorly-differentiated), and the grade correlates with patient prognosis. Poorly differentiated adenocarcinomas tend to be more malignant and prone to recurrence and metastases. The histological grading of patients was statistically significantly different between the two groups. Patients in the non-metformin group compared with those in the metformin group were more likely to have moderately to poorly differentiated tumors, which affects patient prognosis.

In conclusion, tumor diameter, TNM stage, differentiation degree, Ki-67 expression, and pathological subtypes are important indicators of malignancy and prognosis in invasive LUAD. In this study, patients in the metformin group showed advantages over patients in the non-metformin group in all the above indicators. To an extent, metformin affects the invasive and metastatic ability of tumors. These findings suggest a potential association between metformin use and less aggressive tumor features in patients with invasive LUAD. Nevertheless, prospective studies including survival endpoints are needed to validate these observations and determine their clinical relevance.

Given the observed associations between metformin use and more favorable pathological features in LUAD patients with type 2 diabetes, continued use of metformin may be considered in this patient population, in line with current diabetes management guidelines. However, as this was a retrospective study without survival outcomes, we do not recommend initiating metformin solely for oncologic benefit at this stage. Prospective trials are warranted to determine whether metformin should be considered as part of an anti-tumor strategy in this context.

### Limitations and prospects

This study has several limitations. It was a single-center, retrospective analysis with a limited sample size, which may affect the robustness and generalizability of the results. As the study was conducted in a single Chinese hospital, the findings may not be fully generalizable to other healthcare systems. Selection bias and unmeasured confounders—such as diabetes duration, glycemic control, and lifestyle factors—could not be accounted for. In addition, data on HbA1c levels, antidiabetic drug combinations, and treatment duration were unavailable, making it difficult to isolate the independent effect of metformin. The potential confounding effects of concurrent use of other antidiabetic agents (e.g., insulin or DPP-4 inhibitors) could not be assessed. The absence of follow-up information also prevented evaluation of recurrence or survival outcomes.

Nonetheless, our findings are consistent with previous studies suggesting potential anti-tumor effects of metformin in lung cancer. Further prospective, multi-center studies with larger cohorts and comprehensive clinical, metabolic, and survival data are warranted to validate these associations and assess their clinical significance.

## Supporting information

S1 Data Original data set. (XLSX)
